# The Impact of Anthropogenic Climate Change on Egyptian Livestock Production

**DOI:** 10.3390/ani11113127

**Published:** 2021-11-01

**Authors:** Amira A. Goma, Clive J. C. Phillips

**Affiliations:** 1 Faculty of Veterinary Medicine, Alexandria University, Alexandria 21944, Egypt; amira_al2008@yahoo.com or; 2 Curtin University Sustainability Policy Institute, Curtin University, Kent St., Bentley, WA 6102, Australia

**Keywords:** climate change, Egypt, livestock, behaviour, production

## Abstract

**Simple Summary:**

Egypt is one of the hottest countries on the planet, with significant warming predicted to occur over the course of this century. It has a substantial livestock population to feed its growing human population, but the hotter temperatures will constrain the production of ruminants in particular because of their high internal heat production during the digestion of fibrous material by micro-organisms. The net result will be the diminished availability of animal products per human member of the Egyptian population. Some products can be imported, but this is difficult for products with a short shelf life, such as milk. We use estimates of climate change, population growth and the impact of higher temperatures on cow productivity to predict that milk availability per person will decline from 61 kg/year in 2011 to 26 kg/year in 2064. We discuss the range of alternative options available to make up for diminished animal product availability per person as the century progresses.

**Abstract:**

Egypt is one of the hottest countries in the world, and extreme climate events are becoming more frequent, which is consistent with the warming of the planet. The impact of this warming on ecosystems is severe, including on livestock production systems. Under Egyptian conditions, livestock already suffer heat stress periods in summer. The predicted increases in temperature as result of climate change will affect livestock production by reducing growth and milk production because of appetite suppression and conception rate reductions and will increase animal welfare concerns. In severe cases, these effects can result in death. We review the heat stress effects on livestock behaviour, reproduction, and production in the context of predicted climate change for Egypt over the course of this century and offer alternative scenarios to achieve food security for a growing human population. As an example, we combine predictions for reduced milk production during heat stress and human population trajectories to predict that milk availability per person will decline from 61 kg/year in 2011 to 26 kg/year in 2064. Mitigation strategies are discussed and include the substitution of animal-based foods for plant-based foods and laboratory-grown animal products.

## 1. Introduction

The climate of a region is its typical or average weather pattern, and we adopt the climate change definition of being “attributed directly or indirectly to human activity that alters the composition of the global atmosphere and which is in addition to natural climate variability observed over comparable time periods” [1]. This is not an entirely new phenomenon—the average surface temperature of the planet increased by about 1.1 °C in the 19th century. However, climatic extremes have recently increased in both magnitude and frequency [2,3,4,5], with further increases projected in the future [6,7,8].

Mediterranean ecological systems, including the agriculture and water cycles, are being drastically affected by climate change [9], with harmful effects on animal welfare and production, which are expected to increase in the future [10,11]. Egypt has a precarious economy, which depends, in part, on livestock production for income and food security. Even though the efficiency of land and water use is less for animal proteins than it is for plant proteins, those from an animal source are still favoured [12,13]. Food safety and security are key priorities in the coming century, and, with population increases, the demand for food production is also increasing, including demand for that of animal origin. 

Extreme temperatures and low rainfall periods, which lead to drought, primarily affect crop production, but they also impair animal production functions such as milk yield and quality, egg yield, reproductive performance, and the behavioural and immune responses of animals, and they increase the incidence of diseases [14]. Tropical and sub-tropical countries face the biggest challenges to animal productivity from climate change, with reduced growth, reproduction, and milk production, and dramatic changes in physiological functions caused by heat stress [15]. In Egypt, a reduction in animal production by 25% over the course of this century as a result of global climate change is anticipated [16]. The best coping response to these adverse climatic conditions is the development of resilient management strategies and the genetic selection of heat tolerant livestock species [14]. In addition to the direct effects on livestock production, there are indirect effects because climate change and variability affect soil and pasture fertility [17]. 

Livestock themselves contribute to global warming through greenhouse gas (GHG) emissions and accounts for up to 18% of total emissions [18]. Climate change can also have a direct impact on livestock keepers and the goods and services on which they depend [19]. Thus, livestock production is under pressure due to the negative impact of these environmental implications, especially in terms of GHG emissions [20].

Heat stress directly and indirectly affects animal production and welfare. The major indirect effects are a reduction in feed and water intake and feed efficiency, a disturbance in mineral balances, enzymatic activities, and hormonal secretion, all leading to impaired immunity, which, together with reduced pasture availability, leads to more disease outbreaks [20]. In addition, there is also an impairment in productive and reproductive performance [15]. However, there are physiological and biochemical adaptations to heat stress to protect vital cell functions and that allow rapid recovery, especially in the case of moderately hypothermic conditions [21]. 

An industrial-based livestock system is potentially less affected by climate change than mixed farming and grazing systems. In grazing systems, there are direct effects of solar radiation and high temperatures on livestock, and drought causes major reductions in pasture growth [14]. In developing countries, there is a reliance on traditional systems of grazing livestock rather than intensive animal production systems due to a lack of capital for infrastructure [22,23,24]. Several adaptive technologies are being developed to cope with the challenges of climate change, including drought-tolerant fodder crops, the improved use of weather information by livestock keepers to make better management decisions, changes in the livestock species and genotypes used, and policy changes such as market and infrastructural development [19]. 

## 2. Climate Profile of Egypt

For at least the last decade, it has been acknowledged that Egypt is one of the countries that is the most exposed to climate change [25], which is largely due to the extent to which it relies on natural resources. It has been estimated that it is the fifteenth most vulnerable country to human-induced environmental change [26]. Climate change in Egypt is already evident in the form of lower precipitation rates, changing weather patterns, increased CO_2_ in the atmosphere, methane and nitrous emissions, and sea level rise [18]. 

The Egyptian climate is characterized by warm days and cold nights. There are two main seasons: a mild winter (November–April) and a hot summer (May–October). The average temperature range in the coastal regions is between a 14 °C minimum in winter and a 30 °C maximum in summer. In the desert areas, the temperatures reaches 7 °C at night and 43 °C during the day in summer, while in winter, temperatures are as low as 0 °C at night and 18 °C during the day [27]. The annual mean air temperatures range from 20–22 °C in coastal region to 28–30 °C in the south-eastern corner [27].

A Global Rice Science Partnership report [28] compared two periods, from 1901–1950 and from 1951–2016. The report highlighted increases in the maximum temperature, about 0.6 °C and 0.4 °C in winter and summer, respectively, in lower Egypt, which was considered to be primarily due to the growth in urbanization in that region. The minimum temperature increases seen in winter ranged from 0.85 °C in Cairo to 0.50 °C in southern Egypt, and in summer, the increase seen in southern Egypt was 0.7 °C, while in the north, the increase was 0.45 °C. This temperature reversal between the two zones in the two seasons may be due to heat retention in the desert in the south in summer as well as to an absence of wind and rain. 

The annual current average temperatures are 20 °C on the Mediterranean coast, 24 °C on the Red Sea coast, 25 °C in Cairo, and 26 °C in Aswan in the south [29]. The typical maximum temperatures for daytime in midsummer range from 30 °C in Alexandria on the Mediterranean coast to 41 °C in Aswan in the far south, while daytime midwinter maximum temperatures are 18°–23 °C. There was greater warming in summer (0.31 °C/decade) than in winter (0.07 °C/decade) between 1960 and 2003 [29].

Solar radiation (4500 KJ/m^2^) is high during the entire year for all Egyptian zones, with extremes during the summer season and high humidity (50–75%) in the coastal region and low humidity in the very dry desert areas [30]. Farm animals experience climate stress for about 6 months of the year, causing various physiological and biochemical changes due to the increased heat production and reduced heat loss, manifesting in impaired health, production, and reproduction [31,32].

## 3. Climate Change Estimates for Egypt

Early this century, the IPCC predicted a 1.4 to 5.8 °C increase in the average global surface temperature over the course of the 21st century [33]. The Second National Communication (SNC) to the United Nations Framework Convention on Climate Change (UNFCCC) reported that, “Egypt is one of the most vulnerable countries to the potential impacts and risks of climate change” [34]. Climate extremes have occurred frequently during the first decade of this millennium, with estimates of increasing intensity due to climate change. Overall, Mediterranean Africa is likely to be warmer and drier [35].

The anticipated average temperature increase due to global warming is 4 °C for Cairo and 3.1–4.7 °C for other areas of Egypt by 2060 [36]. Temperature change estimations from the records of 11 meteorological stations distributed around the country during the 1960 to 2005 period [27] show that the mean annual temperature increased by 2.1 °C. A warming trend was detected throughout the whole country. Using estimates for the period from 1976 to 2005, an increase in both the daily maximum and minimum temperature has been detected [27]. During the period from 2006–2015, the average maximum temperature increase was from 1.4 to 2.6 °C, while the minimum temperature increase was between 1.2–1.8 °C [8].

One of the most severe heat waves in the Mediterranean area was in June and July 2007 [37], with temperatures reaching 45 °C in northern Egypt [38]. A heat wave is a period of prolonged excessively hot weather that is associated with increased humidity in coastal areas. It is measured in relation to the normal weather of the area and the normal temperature of the season. Heat waves are formed when barometric pressure increases above 3000–7600 m [39] and when it remains high for several days/weeks, which is common in the summer season. In summer, changes in the weather pattern occur more slowly than they do in winter; hence, the high pressure moves away more slowly. Under this high pressure, hot air sinks toward the surface, leading to warming and drying. This warm sinking air inhibits convection, trapping warm humid air underneath, which results in a continuous heat build-up at the surface that is experienced as a heat wave [40]. There have been 12 heat wave occurrences between 1981 and 2012 in Damietta, Egypt [40].

Observations from the Mediterranean and Middle East regions between 1950 and 2000 indicate that these regions warmed faster from June to August than the global average temperature [41]. Therefore, projected temperature increases are likely to be greater in Egypt than they are globally. Over the whole of North Africa, both annual minimum and maximum temperatures are likely to rise in the future, especially the minimum temperature [42]. 

In Egypt, between 1960 and 2010, the maximum and minimum temperatures increased at rates of 0.27 ± 0.03 and 0.30 ± 0.07 °C/decade, respectively. In the period from 1980 to 2017, this rate increased to 0.40 ± 0.1 °C/decade, indicating an acceleration of the warming [8]. The IPCC expect that the sea level will rise by 0.5–1 m by 2100 [43], and the IPCC Fifth Assessment Report predicted an increase in the global average surface temperature of between 0.3 and 4.8 °C by 2100 [44]. Climate change projections expect a temperature increase over Egypt of about 3–3.5 °C and over the Middle East region in general [29]. Additionally, it is predicted that the average annual temperature in Egypt will increase by 1.07–1.27 °C by 2030, with a 10th percentile of 0.37–0.61 °C and a 90th percentile of 1.78–2.11 °C [45]. The projected increase in the maximum temperature is predicted to be 1.00–1.22 °C, and the minimum temperature increase is anticipated to be 1.09–1.32 °C. By 2050 (2040–2059), the mean annual temperature will increase by 1.64–2.33 °C, with a 10th percentile of 0.81–1.52 °C and a 90th percentile of 2.62–3.45 °C [45]. This increase is expected to be greater in summer, and the number of hot days will increase, and the number of cool days decrease. Table 1 and Table 2 give the predicted temperature increases from 2025 to 2100 for high and low emission scenarios [46,47].

Nashwan et al. [48] predicted increases in the maximum (1.80–3.48 °C) and minimum (1.88–3.49 °C) temperatures in the Central North region of Egypt (CNE) by the end of the century. In addition, they anticipated that the annual temperature will increase by an average of 5.6 °C between 1990 and 2100 under a high emissions scenario, with more warm days, increasing from less than 10 days in 1990 to 195 days in 2100. If there are decreased emissions, the temperature increase will be limited to 1.6 °C, and the warm days will be limited to 45 days [49].

Mostafa et al. [8] predicted temperature increases of between 0.28 ± 0.04 °C/decade and 0.38 ± 0.09 °C/decade between 2010–2040 but higher temperature increases between 2050 and 2100, from 0.48 ± 0.18 °C to 0.72 ± 0.11 °C/decade. When compared to the period of 2006–2015, they predicted more frequent hot days and nights, which will occur at a higher rate near the Red Sea and in upper Egypt sites than they will in other areas. In the last decade, 80% of the days/year were hotter than 90% of days in the reference period of 2006–2015. Additionally, in comparison with the reference period from 1986–2005, the temperature increase will be between 0.3- 4.8 °C in 2081–2100. For 2090, it was projected that the maximum temperature will be between 2.0 –0.6 °C and 5.6 ±0.5 °C above that of 2006–2015 values. 

In 2007, the Egyptian Ministry of Water Resources and Irrigation climate change adaptation strategy [50] anticipated that the temperature will increase 1 °C by 2025, which appears likely to be true; similarly, the potential impact of climate change on the Egyptian economy report [51] anticipated an increase between 0.9 and 1 °C by 2030.

## 4. Climate Change Impacts on Livestock Production in Egypt

Livestock production contributes significantly to the national economy of the countries of the Near East and North Africa region, with increases from 186 million to 412 million head over the 40 years until 2010 [52]. Livestock production represents 24.5% of the agricultural Gross Domestic Product (GDP) [53,54]. In 1999, livestock production had contributed 27% of domestic agricultural production [55]. The main livestock production in Egypt is by small-scale farmers, with government farms contributing to less than 2% of the production [54]. Egyptian animal production depends on water buffaloes (70%) and cattle (30%) for annual milk-production and using males and barren females for meat production [56]. The buffalo population increased from 3,250,000 head in 1993 to 4,200,000 head in 2013 [57] due to an increased demand for buffalo products such as milk, cheese, and butter. However, the numbers of buffaloes and cattle have also been estimated as 3.5 and 5.01 million head, respectively, in 2016 [58]. In 2016, the buffalo herd contained 42% dairy buffalo cows, 32% buffalo heifers, 6% bulls, and 20% male calves.

In 2016, poultry meat production in Egypt was about 148,517 metric tons, which exceeded all other livestock meats, including beef and veal, buffalo meat, sheep and goat meat, camel meat, and others. Calf (buffalo and cattle) production increased to 1.93 million head in 2018 from 1.85 million head in 2017 [17]. A similar pattern of poultry dominance has been seen in other countries in the region. In Algeria, chicken production was 137,235 metric tons in 2016, increasing to 138,500 in 2019. At the same time, cattle production, which was from 2,081,306 head in 2016 dropped to 1,780,591 head in 2019. In Tunisia, chicken production was 90,742 metric tons in 2016, increasing to 92,663 metric tons in 2019, while the cattle population was 646,100 head in 2016, increasing to 653,611 head by 2018. In Syria, chicken production was 16,600 metric tons in 2016, increasing to 18,498 in 2019; cattle production was 1,090,000 head in 2016, decreasing to 788,321 head in 2019 [57].

In 2017, the human population was about 104 million, with 56.7% living in rural areas and 43.3% living in urban areas [58]. By 2020, the population had reached 120 million, with the growth rate anticipated to be unchanged. It is expected to increase to 151 million in 2050, with the urban share becoming 56.5% [59]. Although livestock numbers in the last 20 years have increased, this increase is not adequate for the requirements of the increased population, especially in terms of dairy products. Only in the poultry sector has a steady increase in numbers been evident, increasing by about 4% per year [58], which is more than sufficient to cater for the annual human population increase of 1.9%, but this growth may not be sustainable in the future. Numbers of ruminant livestock have declined, and a decrease of about 10% has been seen in sheep and buffalo since about a decade ago, which is probably due to the pressure of urbanization, drought, and diminishing feedstocks. Livestock production (principally from cattle, buffalo, sheep and goats, and camels) contributes to about 37.5% of agriculture production [60]. 

Livestock production and health is challenged both directly and indirectly by climate change. With higher temperatures, livestock health and production are challenged by heat stress, metabolic disorders, oxidative stress, and immune suppression, together with an increased incidence of diseases. The indirect effects are related to the multiplication and distribution of parasites and infectious pathogens and the impact on feed availability [61]. The direct impact of climate change on livestock is influenced by the ability of animals to exchange heat with their environment [53]. If animals lack the ability to dissipate environmental heat, they suffer from heat stress. There are various detrimental effects of heat stress on livestock, with the most important economic impact being on reproduction and milk production in dairy cows. Heat stress has a direct adverse effect on milk production, growth, feed intake, reproductive efficiency, and disease incidence in dairy cows [62]. When the environmental temperature rises above the thermal comfort zone, it adversely affects the growth and productivity of animals in both intensive and extensive production systems. In addition to the reduction in milk production, it leads to a reduction in sperm quantity and quality and a decline in female fertility and embryo quality. 

Heat stress also decreases the body weight of beef cattle due to decreased feed intake and feed conversion efficiency [17]. Additionally, dairy cattle in heat stress have a particular stance, with a lowered head, backward-facing ears, and a vertical tail [63]. An increase in the ambient temperature could also reduce forage digestibility in ruminants because of the greater lignification of the plant [64]. A negative correlation has been found between ambient temperature and degradability, in which the potential degradability of plants was decreased in tropical and arid climates by 0.55% compared to cold and temperate climates, for each 1 °C increase in ambient temperature. 

Farm animals have behavioural strategies to attempt to mitigate the impact of heat stress. Birds respond to heat stress by extending their wings, holding them away from their body, increasing their respiration rate, which has the potential of leading to exhaustion, in an attempt to reduce the heat load through water vapor cooling. Other strategies include looking for isolated places to lower their body temperature, moving away from each other, less activity, decreasing feed intake, and increasing water consumption [65]. The negative effects of climate change on poultry production are mostly direct, reducing weight gain and meat quality in broilers and the rate of egg production, egg quality (especially thin and breakable eggshells), egg weight and size, and a high mortality rate in laying hens [65]. There are also indirect effects reducing growth and reproduction, in particular through the enhanced growth of toxin-producing fungi in feed or by reducing the bird’s ability to cope with other stressors. Moreover, immunity is reduced in birds exposed to heat stress, including a weak immune response to vaccines, which lowers the birds’ resistance to infectious diseases. 

A temperature increase of 1 °C above the ideal temperature leads to a reduction in the feed consumption of laying hens by 1.6%, while the amount of energy consumed reduces by about 2.3% [65]. The egg weight also decreases at rate of 0.07–0.98 g/egg for every 1 °C rise, which can be attributed to the decrease in calcium availability because of the reduction in the food consumption. When temperatures increase to above 30 °C, feed consumption and egg production reduce by 5% and 1.5%, respectively. Approximately one half of this reduction in food consumption occurs when the temperature is elevated from 21 to 38 °C. 

## 5. Climate Change Effects on Milk Production

More than 50% of cattle population is present in the tropics, which explains why heat stress will cause a substantial economic loss in dairy farm profitability around the world [66,67]. At temperatures between 10 and 30 °C, most livestock species perform well. Above this, a reduction in milk yield and feed intake occur in cattle [68,69,70], as, when the rectal temperature of dairy cows exceeds 39.4 °C, the cows need more energy for the maintenance of their body temperature at the expense of energy needed for milk production [71]. Additionally, as previously described, temperatures above 35 °C activate the thermal stress regulatory mechanisms to reduce feed intake, leading to a negative energy balance that affects milk synthesis [72,73]. 

Every unit increase above a temperature–humidity index (THI) value of 71 reduces milk yield by 0.2 kg/day in dairy cows that have no access to shade [71,74,75]. When over 78, milk production also declines in those having access to both shade and sprinklers. In the Mediterranean climate, other researchers have estimated that every unit increase in the THI above 69 reduces milk production by 0.41 kg per cow per day [76,77], but the reduction will depend on the physiological state of the animals and the environment. In farms without sprinklers or other heat mitigating measures, milk yield can decline by 40–50% under heat stress, while in cooled farms, it may only be 10–15% [74,78]. This can mainly be attributed to decreased nutrient intake [77], but Ernabucci and Calamari reported that the reduction in milk yield ranges from 10 to >25%, half of which is due to the reduction in feed intake and the remaining half to heat-related lactogenic hormone reduction [79]. The decrease in milk production is particularly affected by the cow’s breed, age, stage of lactation, and forage availability. An elevated core body temperature not only reduces milk output but also reduces milk protein, fat and lactose percentages in milk, and colostrum [80,81,82]. Furthermore, calcium (Ca), phosphorus (P), and magnesium (Mg) content are reduced in milk, and chloride is increased [83]. There is a reduction in milk short and medium chain (C4–C10, and C12–C16, respectively) fatty acids and an increase in long-chain fatty acids (C17–C18) [83]. This is due to a reduction in their synthesis in the mammary glands and energy being used by the cow to cope with the heat stress. Moreover, α- and β-casein and P in milk are reduced, and the pH increases [84]. 

Heat stress affects prolactin, thyroid hormones, glucocorticoids, growth hormone, estrogen, progesterone, and oxytocin levels, all of which are involved in the control of milk production. In addition, mammary gland involution during the dry period is retarded by heat stress, leading to apoptosis, autophagy, and decreased epithelial cells [82]. Furthermore, heat stress can lead to immunosuppression, with an increase in somatic cell count and more disease in dairy cattle [85]. The mammary gland uses a negative feedback mechanism to reduce milk production [20,86], and if the udder temperature increases, it can also lead to mastitis [87]. Colostrum and IgG transfer to colostrum are also reduced when heat stress occurs near calving time [74,88].

Genetic improvement for increasing milk production is linked to greater feed intake, which makes high yielding cows more sensitive to heat stress than low yielding ones. For high yielding cows, the upper limit of the thermoneutral zone is moved to a lower temperature [89]. 

We reviewed 18 published temperature increase estimates in Egypt (Table 3), which were, on average, from a mean reference year of 2011 over a period of 56.4 years to a mean year of prediction of 2067. Over that same period, the Egyptian population is forecast to increase from 84,529,251 in 2011 to 189,463,934 in 2067 [58]. The temperature –humidity index was calculated for the prediction years. Assuming a conservative 0.21% milk yield reduction per unit of THI increase over 68 [76], we then calculated the THI for the prediction year - 68 and the reduction in milk yield per cow per year at the higher temperature in the year of prediction. This showed that on average, there would be a 3.88% reduction in milk production per cow over the 56.4 years of our survey. We then calculated mean milk consumption per head of the population in Egypt for the year 2011 by taking the mean of years 2017 and 2013 (in kg, 59.46 + 61.81 = 60.64/capita/year [57]). We calculated national milk consumption in 2011 as the Egyptian population (84,529,251) × 60.64 kg/capita/year = 5,125,853,781 kg. We then estimated the milk available for consumption in 2067 as milk consumption in 2011 × 0.9612 (100–3.88%) = 4,926,970,654.2972 kg, which when divided by the population in 2067, gives 26.00 kg/capita/year. Thus, the amount of milk available for consumption in 2067 is predicted to be reduced to 26.00 kg/capita/year from 60.64 kg/capita/year in 2011. 

The options to maintain milk consumption are to (1) increase the national herd size by a factor of 2.64, i.e., from 4.95 million to 13.07 million, keeping the production per cow the same, or (2) to increase production per cow by a similar proportion. Alternatively, milk imports could be increased, or alternative, plant-based milks could be manufactured in Egypt or imported from outside.

## 6. Egypt’s Proposed Mitigation and Adaptation Actions

A decade ago, Egypt announced a national strategy for adaptation to climate change and disaster risk reduction [90], which had three main components: 1. increasing the flexibility of the Egyptian community in dealing with the risks and disasters caused by climate change and its impact on different sectors; 2. enhancing the capacity to absorb and contain climate-related risks and disasters; 3. reduction of climate change-related disasters.

In Egypt’s Third National Communication in 2016 [27], the overall goal of the Egyptian national strategies and programs was to increase livestock production by adaptation to climate change. This addresses both criteria 1 and 2 listed above. It was anticipated that this could be achieved by matching stocking density increases with greater crop production, rotating pastures, modifying grazing times to cooler periods of the day, altering forage and animal species/breeds, and altering the integration of livestock/crop systems, including the use of adapted forage, increased fertilizer application, ensuring an adequate water supply, and the better use of supplementary feeds. It was also intended to improve low-productivity cattle through better feeding programs, enteric fermentation, and manure management [91]. However, biological limits to these factors may already have been reached; hence, other options must be considered. 

Other possibilities include environmental modification through the use of technology to prevent solar heat loads and increasing opportunities for animal heat loss through using shelter and cooling systems; also, nutritional options, such as fibrolytic enzyme supplementation, could be used. Supplementation with exogenous cellulolytic enzymes in ruminant diets can improve the digestibility and feeding value [92]. Ruminants could be supplemented with rice straw and grains inoculated with fungal and bacterial strains to improve digestibility and to reduce methane production. Additionally, more sustainable by-products and novel feeds could be used; including the potential use of tree forages to increase the growth performance of livestock, contributing to food security [93]. However, the selection of the best fodder tree species is important because of the association between the tannin content of fodders with enteric fermentation efficiency and its contribution to climate change mitigation. Paulownia leaves are particularly rich in crude protein, essential amino acids, and phenolic substances [94]. Therefore, supplementation with Paulownia hybrid leaves and ensiled Paulownia hybrid leaves could potentially mitigate methane production and could improve ruminal fermentation. Additionally, they may serve as valuable components in ruminant diets to increase nutrient and energy supply in particular, as well as potentially improving the fatty acid profile of milk. 

Animal selection and breeding must take into account the conflict between those favouring adapted indigenous, low-producing animals and those favouring exogenous high-producing, less tolerant animals. Following the latter strategy will require increased grain feeding at a time when the human population is growing and when food security is therefore declining [95]. Livestock can adapt themselves to low quality crop residues such as straws and stubbles. Therefore, there is an advantageous use for fodder trees, shrubs, and cacti [96]. In ruminant diets that contain triticale and oats as the sole grain sources, a partial replacement for true protein (e.g., soybean meal) with feed-grade urea as a source of nonprotein nitrogen may result in an improvement in microbial protein synthesis in the rumen, minimizing N excretion to the environment [97]. Treating triticale and oat grains with urea can modulate ruminal microbiota and fermentation beneficially, consequently improving production performance and profitability [98].

The development of low-cost feed options, “feed blocks”, that consist of multiple cheap, easily available agro-industrial by-products, such as tomato pulp, molasses, burghul derivatives, crude olive cake, sesame cake, citrus pulp, sunflower cake, and mulberry leaves, will help reduce reliance on grains. Different combinations have been tested in Syria, Iraq, and Tunisia. For example, replacing part of the barley grain component of the diet with molasses and urea in combination with urea-treated straw for feeding ewes improved the reproduction of the ewes, who could be mated earlier, giving birth at shorter intervals, and producing lambs that were heavier at weaning, as a result of increased milk production [99]. These alternative agro-byproducts and ammoniated straws are considered options for poor farmers in the Middle Eastern region to increase their livestock productivity and income without affecting the quality of the products [100].

As ruminants contribute to the predicted temperature increases through greenhouse gases (GHGs), there is pressure to reduce these by improved feeding management, more efficient enteric fermentation, and, in particular, reduced methane emissions. A higher proportion of concentrate can result in a methane emission reduction, and methane emission reduction from enteric fermentation can be achieved by feeding diets containing feed additives, antibiotics, or vaccines. Additionally, good pasture management through rotational grazing is considered an effective way to reduce GHG emissions [101]. Ruminants fed on high fibre and low protein diets produce more methane than those fed on good quality forages or those supplemented with concentrate feed [102]. Therefore, adjusting animal diets can reduce GHG emissions through the better matching of their nutritional requirements with less methane production.

In addition to the biotechnological options, such as the greater use of artificial insemination (AI), embryo transfer (ET), in vitro fertilization (IVF), and sexing and cloning, molecular biotechnology could revolutionise animal health and production using DNA-based applications and enhanced vaccination programs, e.g., against avian flu. In the same context, the feed production gap can be closed through these adaptation options: (i) avoiding feeding berseem clover alone and combining it with lower protein feeds, e.g., grass silage, to avoid wasting protein, which also has a significant detoxification cost in ruminant livestock, (ii) rangeland improvement, (iii) crop residue use, (iv) hydroponic barley feeding, (v) saline water utilization for crop production, and (vi) better livestock integration into the arable farming systems to utilize manure better. If these are not successful, the Egyptian population must rely on alternative strategies to achieve food security, such as plant-based proteins and meat grown in laboratories in vitro. These could be produced more efficiently, depending on whether livestock rely on by-products with no other use. The high cost of in vitro meat production is currently a barrier to widespread adoption [27]. 

## 7. Conclusions

Livestock production in Egypt is likely to face severe challenges if the predicted temperature changes occur later this century, as seems likely from recent experience. As one of the hottest countries on the earth and as a country with a rapidly growing human population, heat stress will become the major constraint on animal production unless there is appropriate mitigation. Mitigation options are available but are expensive to implement and do not seem compatible with the growing needs of the human population for safe, affordable food. Alternative high-value foods, such as clean meat and processed plant proteins will therefore need to be introduced to achieve food security. This will require the re-education of the Egyptian population about the value of alternatives to livestock in their diet and the provision of assistance for Egyptian livestock farmers who are willing to convert to more efficient methods of producing food for a secure future for Egyptians. As ruminants contribute to the predicted temperature increases, a reduction in ruminant livestock use for food production could reduce the anticipated temperature changes. 

## Figures and Tables

**Table 1:** Predicted temperature increases for 2025 to 2100 for a low-emission climate change scenario, and a high-emission climate change scenario, from the First IPCC first Assessment Report for the West, Middle, and East Delta regions of Egypt [46].

Predicted Temperature Increase (°C)				
Year	2025	2050	2075	2100
Low emissions	0.9	1.3	1.8	1.8
High emissions	1.2	2.2	3.2	4.0

**Table 2:** General circulation model estimates of temperature increases in Egypt [47].

Year	Predicted Temperature Increase (°C), Mean and (Standard Deviation)		
	Annual	December/January/ February	June/July/August
2030	1.0 (0.15)	0.8 (0.21)	1.1 (0.18)
2050	1.4 (0.22)	1.2 (0.30)	1.7 (0.26)
2100	2.4 (0.38)	2.1 (0.52)	2.9 (0.45)

^a^ DJF = December, January, and February ^b^ JJA = June, July, and August.

**Table 3:** Review of predictions of increased temperature in Egypt, temperature–humidity index (THI), and reduction in total milk yield (TMY) per cow and available milk yield per person.

Sources for Predicted Temperature Increase	Reference Year for Predicted Increase in Temperature (°C) (x = 2011)	Predicted Increase in Temperature (°C) for Expected Year	No. Years for Temperature Change	Temperature Predicted (°C)	THI	THI-68	% Reduction in TMY (Predicted Decrease for THI Units > 68)
36	2002	4.0 (2060)	58	34	88.36	20.36	4.28
44	2013	4.8 (2100)	87	34.8	89.60	21.6	4.54
29	2013	3.5(2100)	87	33.5	87.58	19.58	4.11
45	2015	1.27 (2030)	15	31.27	84.12	16.12	3.39
45	2015	2.33 (2050)	35	32.33	85.77	17.77	3.73
47	2004	1.2 (2025)	21	31.2	84.01	16.01	3.36
47	2004	2.2 (2050)	46	32.2	85.57	17.57	3.69
47	2004	3.2 (2075)	71	33.2	87.12	19.12	4.02
47	2004	4.0 (2100)	96	34	88.36	20.36	4.28
46	2008	1.0 (2030)	22	31	83.70	15.7	3.3
46	2008	1.4 (2050)	42	31.4	84.32	16.32	3.43
46	2008	2.4 (2100)	92	32.4	85.88	17.88	3.75
49	2015	5.6 (2100)	85	35.6	91.47	23.47	4.93
8	2019	0.38 (2040)	21	30.38	82.74	14.74	3.1
8	2019	0.72 (2100)	81	30.72	83.27	15.27	3.21
8	2019	4.8 (2081–2100)	72	34.8	89.60	21.6	4.54
8	2019	5.6 (2090)	71	35.6	90.84	22.84	4.8
51	2013	1.0 (2025–2030)	14	31	83.70	15.7	3.3

## References

[1] International Plant Protection Convention (IPPC) (2018). Annex I: Glossary. In Global Warming of 1.5 °C; An IPPC Special Report on the Impacts of Global Warming of 1.5 °C above Pre-Industrial Levels and Related Global Greenhouse Gas Emission Pathways, in the Context of Strengthening the Global Response to the Threat of Climate Change, Sustainable Development, and Efforts to Eradicate Poverty; Masson-Delmotte, V., Zhai, P., Pörtner, H.O., Roberts, D., Skea, J., Shukla, P.R., Pirani, A., Moufouma-Okia, W., Péan, C., Pidcock, R., et al., Eds.. https://www.ipcc.ch/site/assets/uploads/sites/2/2019/06/SR15_Full_Report_High_Res.pdf.

[2] Frölicher T.L., Laufkötter C. (2018). Emerging risks from marine heat waves. Nat. Commun..

[3] Wei N., Zhou L., Dai Y., Xia G., Hua W. (2017). Observational evidence for desert amplification using multiple satellite datasets. Sci. Rep..

[4] Nashwan M.S., Shahid S., Abd Rahim N. (2019). Unidirectional trends in annual and seasonal climate and extremes in Egypt. Theor. Appl. Climatol..

[5] Gado T.A., El-Hagrsy R.M., Rashwan I.M.H. (2019). Spatial and temporal rainfall changes in Egypt. Environ. Sci. Pollut. Res. Int..

[6] Beniston M., Stephenson D.B., Christensen O.B., Ferro C.A.T., Frei C., Goyette S., Halsnaes K., Holt T., Jylhä K., Koffi B. (2007). Future extreme events in European climate: An exploration of regional climate model projections. Clim. Chang..

[7] Zhou L. (2016). Desert amplification in a warming climate. Sci. Rep..

[8] Mostafa A.N., Wheida A., El Nazer M., Adel M., El Leithy L., Siour G., Coman A., Borbon A., Magdy A.W., Omar M. (2019). Past (1950–2017) and future (2100) temperature and precipitation trends in Egypt. Weather Clim. Extremes.

[9] Bouras E., Jarlan L., Khabba S., Er-Raki S., Dezetter A., Sghir F., Tramblay Y. (2019). Assessing the impact of global climate changes on irrigated wheat yields and water requirements in a semi-arid environment of Morocco. Sci. Rep..

[10] Parry M.L., Canziani O.F., Palutikof J.P., van der Linden P.J., Hanson C.E., Intergovernmental Panel on Climate Change (IPCC) (2007). Climate change: Impacts, adaptation and vulnerability. Contribution of Working Group II to the Fourth Assessment Report of the Intergovernmental Panel on Climate Change.

[11] Bernabucci U.N., Lacetera L.H., Rhoads R.P., Ronchi B., Nardone A. (2010). Metabolic and hormonal acclimation to heat stress in domesticated ruminants. Animal.

[12] Winrock International (1992). Assessment of Animal Agriculture in Sub-Saharan Africa.

[13] De Boer A.J. (1994). Animal Agriculture in Developing Countries: Technology Dimensions.

[14] Nardone A., Ronchi B., Lacetera N., Ranieri M.S., Bernabucci U. (2010). Effects of climate changes on animal production and sustainability of livestock systems. Livest. Sci..

[15] Habeeb A.A.M., Gad A.E., EL-Tarabany A.A., Atta M.A.A. (2018). Negative effects of heat stress on growth and milk production of farm animals. J. Anim. Husb. Dairy. Sci..

[16] Seguin B., Rowlinson P., Steele M., Nefzaoui A. (2008). The consequences of global warming for agriculture and food production. Livestock and Global Climate Change.

[17] Rabie T.S.K.M., Omran E.E., Negm A.M. (2020). Potential climate change impacts on livestock and food security nexus in Egypt. Climate Change Impacts on Agriculture and Food Security in Egypt.

[18] Attia-Ismail S.A., Omran E.E., Negm A.M. (2020). Influence of climate changes on animal feed production, the problems and the suggested solutions. Climate Change Impacts on Agriculture and Food Security in Egypt.

[19] Thornton P., Herrero M., Ericksen P. (2011). Livestock and Climate Change.

[20] Baumgard L.H., Rhoads R.P., Rhoads M.L., Gabler N.K., Ross J.W., Keating A.F., Boddicker R.L., Lenka S., Sejian V., Sejian V., Naqvi S.M.K., Ezeji T., Lakritz J., Lal R. (2012). Impact of climate change on livestock production. Environmental Stress and Amelioration in Livestock Production.

[21] McMichael A.J., Ando M., Carcavallo R., Epstein P., Haines A., Jendritsky G., Kalkstein L., Kovats S., Odongo R., Patz J. (1996). Climate Change and Human Health: An Assessment by a Task Group on Behalf of the WHO.

[22] Delgado C.L., Rosegrant M., Steinfeld H., Ehui S., Courbois C. (1999). Livestock to 2020: The Next Food Revolution.

[23] Delgado C.L. (2003). Rising consumption of meat and milk in developing countries has created a new food revolution. J. Nutr..

[24] Nardone A. Evolution of livestock production and quality of animal products. Proceedings of the 39th Annual Meeting of the Brazilian Society of Animal Science.

[25] Alavian V., Qaddumi H.M., Dickson E., Diez S.M., Danilenko A.V., Hirji R.F., Puz G., Pizarro C., Jacobsen M., Blankespoor B. Water and Climate Change: Understanding the Risks and Making Climate-Smart Investment Decisions. 2009. World Bank. https://www.preventionweb.net/files/12832_wbwater.pdf.

[26] El-Shahawi M. (2004). Climate Change and Some Potential Impacts on Egypt.

[27] Egyptian Environmental Affairs Agency (EEAA) (2016). Egypt Third National Communication under the United Nations Framework Convention on Climate Change.

[28] GRiSP (Global Rice Science Partnership) (2013). GRiSP in Motion, Annual Report 2012.

[29] UK Met Office (UKMO) (2013). Climate: Observations, Projections and Impacts: Egypt. https://www.metoffice.gov.uk/binaries/content/assets/metofficegovuk/pdf/research/climate-science/climate-observations-projections-and-impacts/uk.pdf.

[30] Marai I.F.M., Habeeb A.A.M., Gad A.E. (2002). Rabbits’ productive and physiological performance traits as affected by heat stress: A review. Livest. Prod. Sci..

[31] Habeeb A.A.M., Abdel-Samee A.M., Kamal T.H. Effect of heat stress, feed supplementation and cooling technique on milk yield, milk composition and some blood constituents in Friesian cows under Egyptian conditions. Proceedings of the 3rd Egyptian–British Conference on Animal, Fish and Poultry Production.

[32] Habeeb A.A.M., Marai I.F.M., Kamal T.H., Phillips C.J.C., Piggins D. (1992). Heat Stress. Farm Animals and the Environment.

[33] Intergovernmental Panel on Climate Change (IPCC) (2001). Climate Change: The Scientific Basis.

[34] Egypt Environmental Affairs Agency (EEAA) (2010). Egypt Second National Communication under the United Nations Framework Convention on Climate Change.

[35] Christensen J.H., Hewitson B., Busuioc A., Chen A., Gao X., Held I., Jones R., Kolli R.K., Kwon W.T., Laprise R., Solomon S., Qin D., Manning M., Chen Z., Marquis M., Averyt K.B., Tignor M., Miller H.L. (2007). Regional climate projections. Climate Change: The Physical Science Basis.

[36] Brauch G.H. (2002). Climate Change, environmental stress and conflict cases of Bangladesh and Egypt. International Conference on Climate Change and Disaster Preparedness.

[37] World Meteorological Organization (WMO) (2008). World Meteorological Organization. Statement on Status of the Global Climate in 2007, WMO-NO. 1031.

[38] Egypt NEWS (2007). Saturday the Peak of a Heat Wave in Egypt. http://news.egypt.com/en/20070730323/news/-egypt-news/saturday-the-peak-of-a-heatwave-in-egypt.html.

[39] Chand R., Ray K., Ray K., Mohapatra M., Bandyopadhyay B.K., Rathore L.S. (2015). Analysis of extreme high temperature conditions over Uttar Pradesh, India. High-Impact Weather Events over the SAARC Region.

[40] International Centre for Agricultural Research in the Dry Areas (ICARDA) (2017). First Report on Climatology of Nile Delta, Egypt; Zaki, A., Swelam, A., Eds.. https://repo.mel.cgiar.org/handle/20.500.11766/6384.

[41] Van Oldenborgh G.J., Drijfhout S.S., van Ulden A.P., Haarsma R., Sterl A., Severijns C., Hazeleger W., Dijkstra H.A. (2009). Western Europe is warming much faster than expected. Clim. Past..

[42] Intergovernmental Panel on Climate Change (IPCC) (2014). Fifth Assessment Report by Working Group II. Climate Change 2014: Impacts, Adaptation, and Vulnerability. Chapter 22 (Africa).

[43] UNDP (2011). Egypt’s National Strategy for Adaptation to Climate Change and Disaster Risk Reduction.

[44] Stocker T.F., Qin D., Plattner G.K., Tignor M., Allen S.K., Boschung J., Nauels A., Xia Y., Bex V., Midgley P.M., Intergovernmental Panel on Climate Change (IPCC) (2013). Climate change: The physical science basis. Contribution of Working Group I to the Fifth Assessment Report of the IPCC.

[45] (2015). World Bank Climate Change Knowledge Portal. https://climateknowledgeportal.worldbank.org/.

[46] Elshinnaway I.A. (2008). Coastal Vulnerability to Climate Changes and Adaptation Assessment for Coastal Zones of Egypt.

[47] Agrawala S., Moehner A., El Raey M., Conway D., Van Aalst M., Hagenstad M., Smith J., Development and Climate Change in Egypt: Focus on Coastal Resources and the Nile (2004). Organization for Economic Co-operation and Development. https://www.oecd.org/env/cc/33330510.Pdf.

[48] Nashwan M.S., Shahid S., Chung E.S. (2020). High-resolution climate projections for a densely populated Mediterranean region. Sustainability.

[49] World Health Organization (WHO) Climate and Health Country Profile—Egypt-2015. https://www.who.int/globalchange/resources/country-profiles/PHE-country-profile-Egypt.pdf?ua=1.

[50] Intergovernmental Panel on Climate Change (IPCC) (2007). Climatic Change: The Physical Science Basis.

[51] Smith J., Deck L., McCarl B., Kirshen P., Malley J., Abdrabo M. (2013). Potential Impacts of Climate Change on the Egyptian Economy.

[52] Food and Agriculture Organisation (FAO) (2011). The State of the World’s Land and Water Resources for Food and Agriculture (SOLAW)—Managing Systems at Risk.

[53] Khelifa H.H. (2012). Climate change risk management in Egypt. Assessment of Climate Change Impacts on Livestock.

[54] Sustainable Agricultural Development Strategy (SADS) (2009). Sustainable Agricultural Development Strategy towards 2030.

[55] Central Agency for Public Mobilization and Statistics (CAPMS) (2000). Statistical Yearbook.

[56] Arefaine H., Kashwa M. (2015). A review on strategies for sustainable buffalo milk production in Egypt. J. Biol. Agric. Healthc..

[57] FAOSTAT (2018). FAO Statistics Division.

[58] (2019). United Nations, Department of Economic and Social Affairs, Population Dynamics. World Population Prospects. https://population.un.org/wpp.

[59] FAO (2017). Africa Sustainable Livestock 2050-. Country Brief.

[60] FAO (2020). The Long-Term Future of Livestock and Fishery in Egypt—Production Targets in the FACE of Uncertainty.

[61] Ali M.Z., Carlile G., Giasuddin M. (2020). Impact of global climate change on livestock health: Bangladesh perspective. Open Vet. J..

[62] Biswal J., Vijayalakshmy K., Rahman H. (2020). Seasonal Variations and Its Impacts on Livestock Production Systems with a Special Reference to Dairy Animals: An Appraisal. Vet. Sci. Res..

[63] Idrus M. (2020). Behavioural and Physiological Responses of Beef Cattle to Hot Environmental Conditions. Ph.D. Thesis.

[64] Moyo M., Nsahlai I. (2021). Consequences of Increases in Ambient Temperature and Effect of Climate Type on Digestibility of Forages by Ruminants: A Meta-Analysis in Relation to Global Warming. Animals.

[65] Soliman A., Safwat A.M., Omran E.E., Negm A.M. (2020). Climate change impact on immune status and productivity of poultry as well as the quality of meat and egg products. Climate Change Impacts on Agriculture and Food Security in Egypt.

[66] Wolfenson D., Roth Z., Meidan R. (2000). Impaired reproduction in heat-stressed cattle: Basic and applied aspects. Anim. Reprod. Sci..

[67] Pegorer M.F., Vasconcelos J.L., Trinca L.A., Hansen P.J., Barros C.M. (2007). Influence of sire and sire breed (Gyr versus Holstein) on establishment of pregnancy and embryonic loss in lactating Holstein cows during summer heat stress. Theriogenology.

[68] Collier R.J., Zimbelman R.B., Rhoads R.P., Rhoads M.L., Baumgard L.H. A re-evaluation of the impact of temperature humidity index (THI) and black globe humidity index (BGHI) on milk production in high producing dairy cows. Proceedings of the 24th Annual Southwest Nutrition and Management Conference.

[69] Allen J.D., Anderson S.D., Collier R.J., Smith J.F. Managing heat stress and its impact on cow behavior. Proceedings of the Western Dairy Management Conference.

[70] Thornton P.K., Boone R.B., Villegas J.R. (2015). Climate Change Impacts on Livestock.

[71] Hassanein M.K., Medany M.A. The impact of climate change on production of maize (*Zea mays* L.). Proceedings of the International Conference on Climate Change and their Impacts on Coastal Zones and River Deltas: Vulnerability, Mitigation and Adaptation.

[72] Wheelock J.B., Rhoads R.P., VanBaale M.J., Sanders S.R., Baumgard L.H. (2010). Effects of heat stress on energetic metabolism in lactating Holstein cows. J. Dairy Sci..

[73] Das R., Sailo L., Verma N., Bharti P., Saikia J., Imtiwati, Kumar R. (2016). Impact of heat stress on health and performance of dairy animals: A review. Vet. World.

[74] West J.W. (2003). Effects of heat-stress on production in dairy cattle. J. Dairy Sci..

[75] Ravagnolo O., Misztal I., Hoogenboom G. (2000). Genetic component of heat stress in dairy cattle, development of heat index function. J. Dairy Sci..

[76] Bouraoui R., Lahmar M., Majdoub A., Djemali M., Belyea R. (2002). The relationship of temperature-humidity index with milk production of dairy cows in a Mediterranean climate. Anim. Res..

[77] Gantner V., Mijic P., Kuterovac K., Solic D., Gantner R. (2011). Temperature-humidity index values and their significance on the daily production of dairy cattle. Mljekarstvo.

[78] Baumgard L.H., Wheelock J.B., Shwartz G., Brien M.O., van Baale M.J., Collier R.J., Rhoads M.L., Rhoads R.P. Effects of heat stress on nutritional requirements of lactating dairy cattle. Proceedings of the 5th Annual Arizona Dairy Production Conference.

[79] Ernabucci U., Calamari L. (1998). Effect of heat stress on bovine milk yield and composition. Zootec. Nutr. Anim..

[80] Ozrenk E., Inci S.S. (2008). The effect of seasonal variation on the composition of cow milk in Van Province. Pak. J. Nutr..

[81] Key N., Sneeringer S., Marquardt D. (2014). Climate Change, Heat Stress and U.S. Dairy Production. A Report Summary from the Economic Research Service, United States Department of Agriculture. http://www.ers.usda.gov/media/1679930/err175.pdf.

[82] Prathap P., Archana P.R., Aleena J., Sejian V., Krishnan G., Bagath M., Manimaran A., Beena V., Kurien E.K., Varma G. (2017). Heat stress and dairy cow: Impact on both milk yield and composition. Int. J. Dairy Sci..

[83] Bernabucci U., Basiricò L., Morera P. (2013). Impact of hot environment on colostrum and milk composition. Cell. Mol. Biol..

[84] Kume S., Takahashi S., Kurihara M., Aii T. (1989). The effects of a hot environment on the major mineral content in milk. Jpn. J. Zootech. Sci..

[85] Temple D., Bargo F., Mainau E., Ipharraguerre I., Manteca X. (2015). Heat stress and efficiency in dairy milk production: A practical approach. The farm animal welfare fact sheet no. 12, Farm Animal Welfare Education Centre. http://www.fawec.org/media/com_lazypdf/pdf/fs12-en.pdf.

[86] Silanikove N., Shapiro F., Shinder D. (2009). Acute heat stress brings down milk secretion in dairy cows by up-regulating the activity of the milk-borne negative feedback regulatory system. BMC Physiol..

[87] Igono M.O., Johnson H.D., Steevens B.J., Hainen W.A., Shanklin M.D. (1988). Effect of season on milk temperature, milk growth hormone, prolactin and somatic cell counts of lactating cattle. Int. J. Biometeorol..

[88] Daramola J.O., Abioja M.O., Onagbesan O.M., Sejian V., Naqvi S.M.K., Ezeji T., Lakritz J., Lal R. (2012). Heat stress impact on livestock production. Environmental Stress and Amelioration in Livestock Production.

[89] Kadzere C.T., Murphy M.R., Silanikove N., Maltz E. (2002). Heat stress in lactating dairy cows: A review. Livest. Prod. Sci..

[90] Information and Decision Support Center (IDSC) (2011). The Egyptian Cabinet and the UNDP: National Strategy for Adaptation to Climate Change and Disaster Risk Reduction.

[91] World Bank Group (2016). Egypt: Intended Nationally Determined Contribution. http://spappssecext.worldbank.org/sites/indc/PDF_Library/EG.pdf.

[92] Zayed M.S., Szumacher-Strabel M., El-Fattah D.A.A., Madkour M.A., Gogulski M., Strompfová V., Cieślak A., El-Bordeny N.E. (2020). Evaluation of cellulolytic exogenous enzyme-containing microbial inoculants as feed additives for ruminant rations composed of low-quality roughage. J. Agric. Sci..

[93] Kitalyi A., Rubanza C., Komwihangilo D., Rowlinson P., Steele M., Nefzaoui A. (2008). Agroforestry and livestock: Adaptation/mitigation strategies in agro-pastoral farming systems of Eastern Africa. Livestock and Global Change, Proceedings of an International Conference, Hammamet, Tunisia, 17–20 May 2008.

[94] Huang H., Szumacher-Strabel M., Patra A.K., Ślusarczyk S., Lechniak D., Vazirigohar M., Varadyova Z., Kozłowska M., Cieślak A. (2021). Chemical and phytochemical composition, in vitro ruminal fermentation, methane production, and nutrient degradability of fresh and ensiled Paulownia hybrid leaves. Anim. Feed Sci. Technol..

[95] Phillips C.J.C. (2015). The Animal Trade.

[96] Salem H.B., Smith T., Rowlinson P., Steele M., Nefzaoui A. (2008). Feeding strategies to alleviate negative impacts of drought on ruminant production. Livestock and Global Change, Proceedings of an International Conference, Hammamet, Tunisia, 17–20 May 2008.

[97] Sinclair L.A., Blake C.W., Griffin P., Jones G.H. (2012). The partial replacement of soyabean meal and rapeseed meal with feed grade urea or a slow-release urea and its effect on the performance, metabolism and digestibility in dairy cows. Animal.

[98] Libera K., Szumacher-Strabel M., Vazirigohar M., Zieliński W., Lukow R., Wysocka K., Kołodziejski P., Lechniak D., Varadyova Z., Patra A.K. (2021). Effects of feeding urea-treated triticale and oat grain mixtures on ruminal fermentation, microbial population, and milk production performance of mid lactation dairy cows. Ann. Anim. Sci..

[99] Tibbo M., Iñiguez L., Rischkowsky B. (2008). Livestock Research for Climate Change Adaptation.

[100] Hilali M., Iñiguez L., Mayer H., Knaus W., Schreiner M., Zaklouta M., Wurzinger M., Solh M., Saxena M.C. (2010). New feeding strategies for Awassi sheep in drought affected areas and their effect on product quality. Food Security and Climate Change in Dry Areas, Proceedings of an International Conference, Amman, Jordan, 1–4 February 2010.

[101] FAO (2009). The state of food and agriculture. Livestock in the Balance.

[102] Pilling P., Hoffmann I. (2011). Climate Change and Animal Genetic Resources for FOOD and agriculture: State of Knowledge, Risks and Opportunities.

